# Overexpression of *Fgf18* in cranial neural crest cells recapitulates Pierre Robin sequence in mice

**DOI:** 10.3389/fcell.2024.1376814

**Published:** 2024-04-17

**Authors:** Yi Lv, Qian Wang, Chensheng Lin, Xi Zheng, Yanding Zhang, Xuefeng Hu

**Affiliations:** Fujian Key Laboratory of Developmental and Neural Biology, College of Life Sciences, Fujian Normal University, Fuzhou, China

**Keywords:** FGF18, cranial neural crest cells, cleft palate, mandible, condylar cartilage, Pierre Robin sequence

## Abstract

The pivotal role of FGF18 in the regulation of craniofacial and skeletal development has been well established. Previous studies have demonstrated that mice with deficiency in *Fgf18* exhibit severe craniofacial dysplasia. Recent clinical reports have revealed that the duplication of chromosome 5q32-35.3, which encompasses the *Fgf18* gene, can lead to cranial bone dysplasia and congenital craniosynostosis, implicating the consequence of possible overdosed FGF18 signaling. This study aimed to test the effects of augmented FGF18 signaling by specifically overexpressing the *Fgf18* gene in cranial neural crest cells using the *Wnt1-Cre;pMes-Fgf18* mouse model. The results showed that overexpression of *Fgf18* leads to craniofacial abnormalities in mice similar to the Pierre Robin sequence in humans, including abnormal tongue morphology, micrognathia, and cleft palate. Further examination revealed that elevated levels of *Fgf18* activated the Akt and Erk signaling pathways, leading to an increase in the proliferation level of tongue tendon cells and alterations in the contraction pattern of the genioglossus muscle. Additionally, we observed that excessive FGF18 signaling contributed to the reduction in the length of Meckel’s cartilage and disrupted the development of condylar cartilage, ultimately resulting in mandibular defects. These anomalies involve changes in several downstream signals, including Runx2, p21, Akt, Erk, p38, Wnt, and Ihh. This study highlights the crucial role of maintaining the balance of endogenous FGF18 signaling for proper craniofacial development and offers insights into potential formation mechanisms of the Pierre Robin sequence.

## 1 Introduction

Normal craniofacial development is a precise process that involves the spatiotemporal coordination of multiple cellular signals, and any interruption of this process may result in abnormal craniofacial structures (Li et al., 2022). Pierre Robin sequence (PRS) is a congenital craniofacial defect, estimated to occur in approximately 1:8500 births, and is typified by mandibular hypoplasia, glossoptosis, cleft palate, and upper airway obstruction (Tan et al., 2013). These structural anomalies contribute to various degrees of airway obstruction and feeding difficulties, which are concomitant with a high mortality rate in infancy (Hsieh and Woo, 2019). The mandibular compression theory suggests that mandibular growth restriction inhibits the downward and forward movement of the tongue, thereby hindering the lift and fusion of the palatal shelves, which is considered a major cause of PRS (Giudice et al., 2018).

The fibroblast growth factor (FGF) signaling plays an important role in craniofacial development by influencing cellular proliferation, differentiation, and survival via multiple signaling cascades, such as RAS-MAPK, P13K-AKT, and PLCγ-PKC (Lee et al., 2018). Studies have shown that mutations in genes encoding FGFR are associated with craniosynostosis in patients (Moosa and Wollnik, 2016). In addition to FGFR, FGF2, FGF3, FGF4, and FGF9 also play a critical role during cranial suture development (Zhao et al., 2023). The loss of FGF10 function affects the process of palatal closure, resulting in abnormal tongue morphology and cleft palate (Prochazkova et al., 2018). Genome-wide analysis has shown that mutations in *Fgf8* were associated with the development of cleft lip and palate (Hao et al., 2019). Further studies have shown that *Fgf8* has dose-dependent effects on jaw shape, size, and symmetry (Zbasnik et al., 2022).

FGF18 is a member of the FGF8 subfamily and is highly conserved between humans and mice. Studies have shown that *Fgf18* ^−/−^ mice survived embryonic development but died in early neonatal life, meanwhile, mostly exhibiting cleft palate and mandibular malformations (Liu et al., 2002). Specific inactivation of *Fgf18* in neural crest-derived craniofacial mesenchyme results in mandibular shortening and reduced ossification of the frontal, nasal, and anterior cranial base skeletal elements (Yue et al., 2021). This piece of evidence suggests that *Fgf18* expression in neural crest-derived mesenchyme plays a critical role in mandibular and multi-craniofacial bone development. Further studies have shown that FGF18 is necessary for cell proliferation and differentiation during osteogenesis and chondrogenesis (Liu et al., 2002; Ohbayashi et al., 2002). Of note, targeting *Fgf18* in calvarial bone development delayed the process of suture closure (Ohbayashi et al., 2002). It has been demonstrated that FGF18 signals partly via FGFR3 to promote osteogenesis and chondrogenesis (Davidson et al., 2005). We accessed the ClinVar database and identified a duplication of chromosome 5q32-35.3, which encompasses the FGF18 gene and causes cranial bone dysplasia and congenital craniosynostosis, implicating a possible consequence of overdosed FGF18 signaling.

In this study, we used a conditionally activated *Fgf18* mouse model to investigate the possible effects of overdosed *Fgf18* in craniofacial bone development. We activated a conditional *Fgf18* transgenic allele in cranial neural crest cells (CNCCs) by crossing *Wnt1-Cre* mice with *pMes-Fgf18* mice generated in our laboratory. Our results demonstrated that *Wnt1-Cre;pMes-Fgf18* mice exhibited the characteristic features of the PRS, such as cleft palate, abnormal tongue placement, micrognathia, and skull malformations. Additionally, we have provided evidence that maintaining a proper balance of endogenous FGF18 signaling is crucial for normal craniofacial development. Dysregulation of FGF18 signals alters the downstream signaling pathways such as Akt, Erk, p38, β-catenin, Ihh, Runx2, and p21, thus affecting the normal proliferation and ossification of craniofacial cells.

## 2 Materials and methods

### 2.1 Animals

The *pMes-Fgf18* transgenic mice were produced by pronuclear injection of the *pMes-Ires-Egfp* vector, which contains the complete cDNA of mouse *Fgf18*. This transgenic vector was created by integrating the *Fgf18* sequence between a *LoxP*-flanked STOP cassette controlled by the *β-actin* promoter and the *Ires-Egfp* sequences. The *Wnt1-Cre* (JAX Stock No. 007807) and *R26R* ^*mTmG*^ (JAX Stock No.007576) transgenic mice used in this study were obtained from the Jackson Laboratory and have been described in previous reports (Zhang et al., 2021). To specifically activate the *Fgf18* transgenic allele in the neural crest-derived mesenchyme, we utilized *Wnt1-Cre* mice and bred them with *pMes-Fgf18* mice to generate *Wnt1-Cre;pMes-Fgf18* mice. Subsequently, these mice were mated with *pMes-Fgf18;R26R* ^*mTmG*^ mice to obtain *Wnt1-Cre;pMes-Fgf18;R26R* ^*mTmG*^ mice. These experimental procedures were approved by the Institutional Animal Care and Use Committee at Fujian Normal University.

### 2.2 Organ culture of palates

For *in vitro* palate fusion assay, paired palatal shelves were carefully dissected from E13.5 *Wnt1-Cre;pMes-Fgf18* mutant and wild-type embryos. Paired palatal shelves were placed on a filter paper in Trowell-type organ culture and were oriented and juxtaposed with the MEE facing each other closely, as described previously (Chen et al., 2019). Samples were cultured in DMEM culture medium containing 20% fetal bovine serum, and incubated at 37° and 5% CO_2_ for 72 h. The medium was changed every 24 h in culture. After culture, samples were collected for fixation and histological analysis.

### 2.3 Rotational explant culture

E13.5 embryos of *Wnt1-Cre;pMes-Fgf18* mutant and wild-type mice were collected and decapitated in sterile cold PBS. The heads, minus the tongue and mandible, were placed in a 20-mL glass bottle filled with 2 mL of DMEM medium supplemented with 20% fetal calf serum. Samples were incubated on a rotary apparatus rotating at a speed of 4 rpm in a vertical position for 24 h at 37°C and 5% CO_2_. After this period, the samples were washed with PBS for fixation and histological analysis.

### 2.4 Skeletal staining

The skulls of *Wnt1-Cre;pMes-Fgf18* and control mice were fixed in 100% ethanol for 1-2 days and soaked in acetone for 3 days. Samples were then washed three times with water and transferred to the Alcian blue-alizarin red mixture for 5 days. Then soaked samples with 95% alcohol solution for 30 min, followed by 2% KOH for hydrolysis and gradient glycerol clearing.

### 2.5 Histology and immunofluorescence

Mouse embryos were harvested from timed pregnant females. Skulls of collected embryos were fixed in 4% Paraformaldehyde at 4°C for 24 h, then decalcified with formic acid for 14 days. Heads were sectioned (7 μm thickness) and stained with hematoxylin and eosin (H&E) or Azan. Immunofluorescence was performed in PBS/10% bovine serum albumin using the following antibodies: anti-Runx2 (Abcam,ab76956), anti-Sox9 (Abcam, ab3697; 1:50), anti-Ki67 (BD, 550609; 1:50), anti-Sp7/Osterix (Abcam, ab22552; 1:100), anti-Caspase3 (Bioss, bs-0081R), anti-GFP (Abcam, ab13970), anti-COLX (Abcam, ab58632), anti-COLII (Abcam, ab34712), anti-Caspase3 (Abcam, ab44976), anti-Scleraxis (Santa Cruz, sc-518082), Anti-FGF18 (Santa Cruz, sc-393471), anti-Collagen Type I (Proteintech, 14695-1-AP), anti-IHH (Proteintech, 13388-1-AP), anti-β-catenin (CST, #8480), anti-pErk1/2 (CST, #4370), anti-pP38 (CST, #4511), anti-pJNK (Santa Cruz, sc-6254). For immunofluorescence staining, an appropriate secondary antibody conjugated to a fluorescence probe was added. This was then incubated at room temperature for 1 h, followed by rinsing in PBS. Finally, the samples were mounted in an anti-fading mounting media. Results were obtained using an Olympus BX51 upright microscope (Olympus Optical, Tokyo, Japan).

### 2.6 *In situ* hybridization

Whole-mount *in situ* hybridization was performed according to published protocols (Zhang et al., 2021). At least three embryos of each genotype were hybridized to the *Shh* probe and only probes that detected consistent patterns of expression in all samples were considered valid results.

### 2.7 Statistical analysis

Data are expressed as the mean ± standard deviation (SD). A comparison of means between the two groups was performed using the Student’s t-test. Differences between multiple groups were compared using one-way ANOVA. *p* < 0.05 was considered statistically significant.

## 3 Results

### 3.1 *Fgf18* overexpression in CNCCs causes severe craniofacial defects

Expression of *Fgf18* has been documented in the paraxial mesoderm of mice at E8.0 (Maruoka et al., 1998; Hagan et al., 2019). To explore the function of FGF18 in craniofacial development, we conducted an analysis of FGF18 expression in the developing palate and mandibular condyle at E13.5 to E18.5 mice. The results have revealed that FGF18 was weakly expressed in the anterior, middle, and posterior regions of the palatal shelves at E13.5 (Supplementary Figures S1A–C). At E14.5, the expression of FGF18 remained at a low level in these regions (Supplementary Figures S1D–F). However, at E15.5, a relatively higher expression level of FGF18 was observed in the palatal shelves (Supplementary Figures S1G–I). By E16.5, FGF18 expression was dramatically increased (Supplementary Figures S1J–L). Additionally, FGF18 was expressed at the onset of condyle primordium formation in E14.5. From E15.5 to E18.5, the expression of FGF18 was restricted to the fibrous/polymorphic progenitor cell layer of the condyle (Supplementary Figures S1M–P). These results suggest that the FGF18 signaling pathway is involved in the regulation of the development of the palate and mandibular condyle.

**FIGURE 1:**
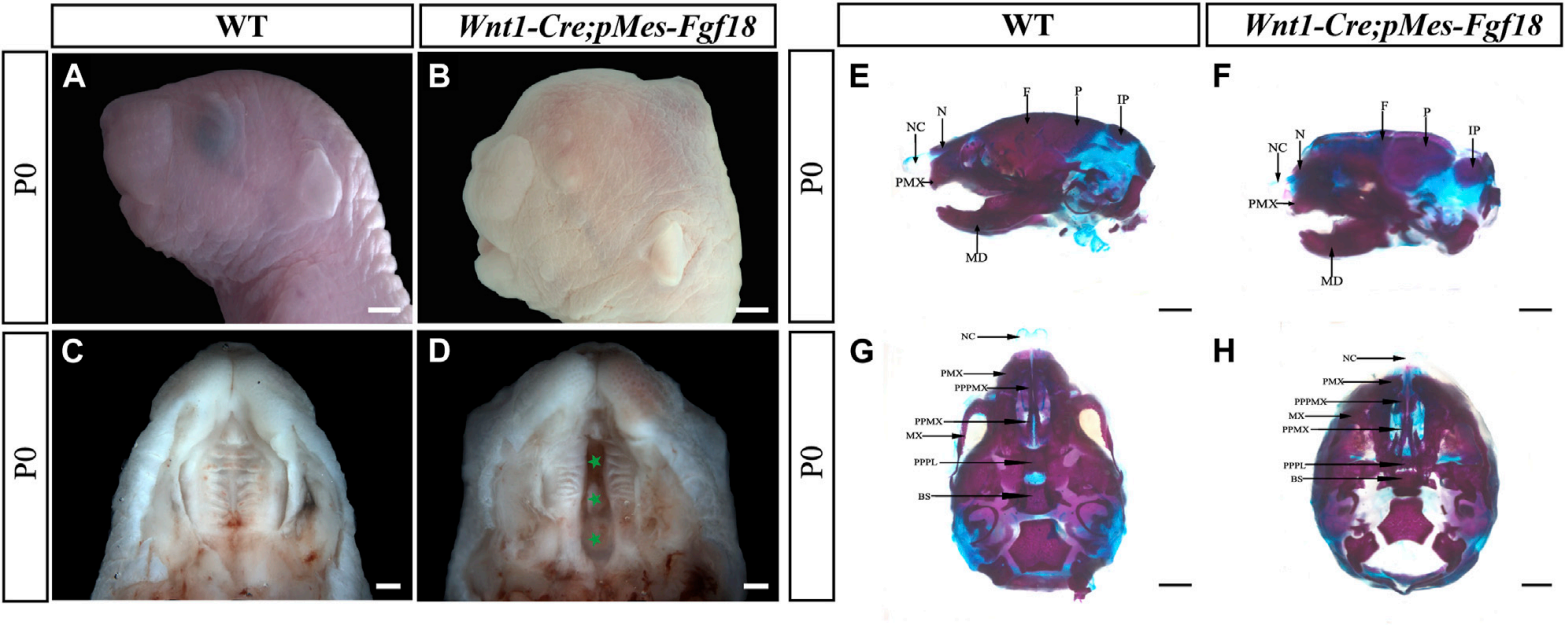
Gross craniofacial phenotype of *Wnt1-Cre;pMes-Fgf18* mice. **(A,B)** Lateral view of the mouse’s head at P0. **(C,D)** Intraoral view of the mice palate at P0. **(E,F)** Lateral view of the mice heads at P0 staining by Alcian Blue-Alizarin Red. **(G,H)** Top view of the mice head at P0 staining by Alcian Blue-Alizarin Red. N, nasal; NC, nasal capsule; P, parietal bone; F, frontal bone; IP, interparietal; MD, mandible; PMX, premaxilla; BS, basisphenoid; MX, maxilla; PPMX, the palatal process of maxilla; PPPL, the palatal process of palatine; PPPMX, the palatal process of the premaxilla. Scale bar = 1,000 μm **(A,B,E–H)**; Scale bar = 500 μm **(C,D)**.

Previous studies have shown that *Fgf18* ^*−/−*^ mutant mice die shortly after birth, most with severe craniofacial deformities such as skull bone defects, micrognathia, and cleft palates (Liu et al., 2002; Ohbayashi et al., 2002). To gain deeper insights into the impacts of upregulated FGF18 signaling in craniofacial development, we employed the *Wnt1-Cre* allele to activate a conditional *Fgf18* transgenic allele in CNCCs by compounding it with the *pMes-Fgf18* allele (Supplementary Figure S2A). The fluorescence analysis of *Wnt1-Cre; R26R* ^*mTmG*^ mice revealed specific expression of Cre recombinase in CNCCs, indicating precise targeting of the transgene in the desired cell population (Supplementary Figures S2B, C). Meanwhile, results from E10.5 *Wnt1-Cre;pMes-Fgf18* showed elevated FGF18 in the branchial arch, verifying successful overexpression of the *Fgf18* allele in CNCCs (Supplementary Figures S2D, E). Intriguingly, *Wnt1-Cre;pMes-Fgf18* mouse embryos also died at birth and exhibited multiple craniofacial malformations, including micrognathia and cleft palate (Figures 1A–D). Skeletal preparations of *Wnt1-Cre;pMes-Fgf18* mice at P0 further confirmed severe craniofacial bone defects, including significantly shortened basisphenoid, maxillary, and mandibular bones (Figures 1E–H).

**FIGURE 2:**
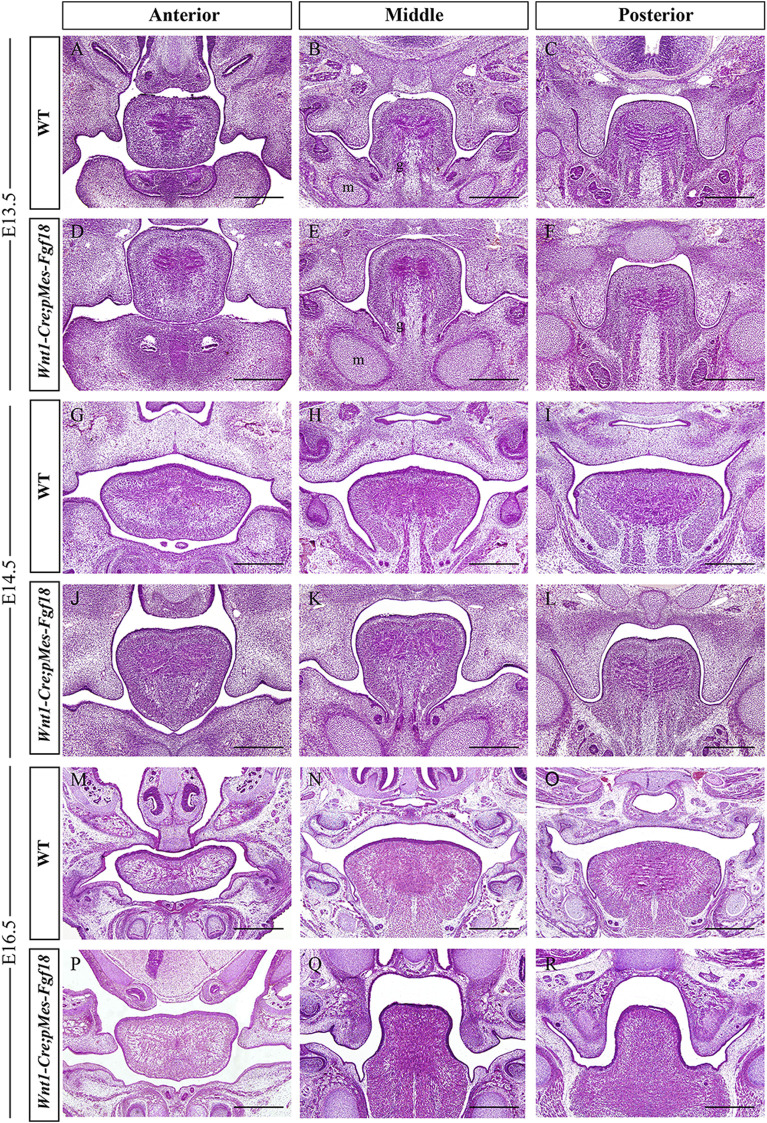
*Wnt1-Cre;pMes-Fgf18* mouse embryos exhibited cleft palate. **(A–I)** Hematoxylin and eosin (HE) stained coronal sections of control and *Wnt1-Cre;pMes-Fgf18* mouse embryos at E13.5 **(A–F)**, E14.5 **(G–L)**, and E16.5 **(M–R)**. m: Meckel cartilage, g: genioglossus, Scale bar = 400 μm.

To understand the developmental changes of the palatal shelves, we examined the histology of the developing palatal shelves in the transgenic mice. Histological analyses revealed that *Wnt1-Cre;pMes-Fgf18* embryos exhibited a similar localization of the palatal shelves at E13.5 compared to control littermates (Figures 2A–F). However, at E14.5, while palatal shelves had already elevated and initiated fusion in control littermates, *Wnt1-Cre;pMes-Fgf18* mouse embryos still had vertically oriented palatal shelves with the tongue wedged in between (Figures 2G–L). Furthermore, all *Wnt1-Cre;pMes-Fgf18* mouse embryos examined from E16.5 to P0 exhibited a failure of palatal shelf elevation (Figures 2M–R). Collectively, conditional *Fgf18* overexpression in the CNCCs disrupted craniofacial development and affected the elevation of palatal shelves, resembling the phenotype of human PRS.

### 3.2 *Fgf18* overexpression altered the direction of genioglossus muscle contraction

**FIGURE 3:**
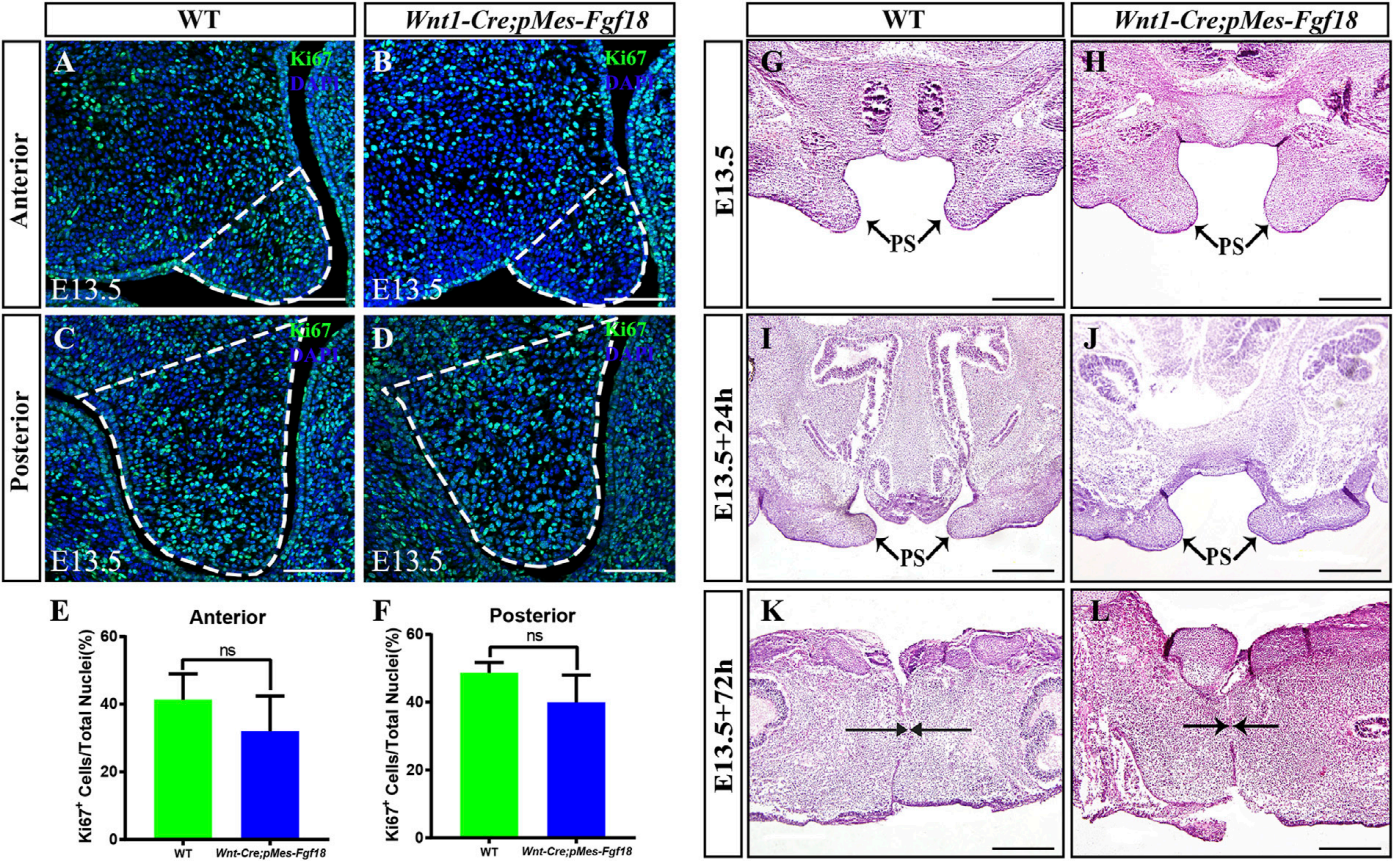
Overexpression of *Fgf18* inhibited the elevation and fusion of palate. **(A–D)** Immunostaining with antibodies against Ki67 was performed on the anterior and posterior regions of mouse embryos palate at E13.5. **(E, F)** Quantification analysis of the percentage of Ki67-positive cells in the anterior and posterior of palate. **(G–L)** After 24 h and 72 h of organ culture, the palatal shelve of E13.5 mouse embryos was stained with hematoxylin and eosin (HE). Scale bar = 100 µm **(A–D)**, Scale bar = 400 µm **(G–J)**, Scale bar = 200 µm **(K, L)**.

FGF transduction signals regulate diverse cellular activities, including cell proliferation and survival (Xie et al., 2020). To investigate whether the cleft palate in *Wnt1-Cre;pMes-Fgf18* mice is caused by changes in cell proliferation and survival, we examined the expression of Ki67 and Caspase3 in the palatal shelves. However, no significant difference was observed in the number of Ki67^+^ and Caspase3^+^ cells within the palatal shelves (Figures 3A–F) (Supplementary Figure S3). These results suggest that the overexpression of *Fgf18* in the CNCCs does not lead to cleft palate by influencing the proliferation and apoptosis of palatal frame cells. However, the cleft palate defects in mouse models are often secondary to defects in tongue movement and mandible. To investigate whether the failure of palatal shelves elevation in *Wnt1-Cre;pMes-Fgf18* embryos were associated with mandible and tongue malformations, we established organotypic cultures of embryos after removing the mandible and tongue. Our findings revealed that the palatal shelves from both E13.5 control and *Wnt1-Cre;pMes-Fgf18* mice were able to elevate and fuse after 72 h in culture (Figure 3G–L). These results suggested that the cleft palate observed in *Wnt1-Cre;pMes-Fgf18* mice is due to physical obstruction caused by the malformed mandible/tongue.

**FIGURE 4:**
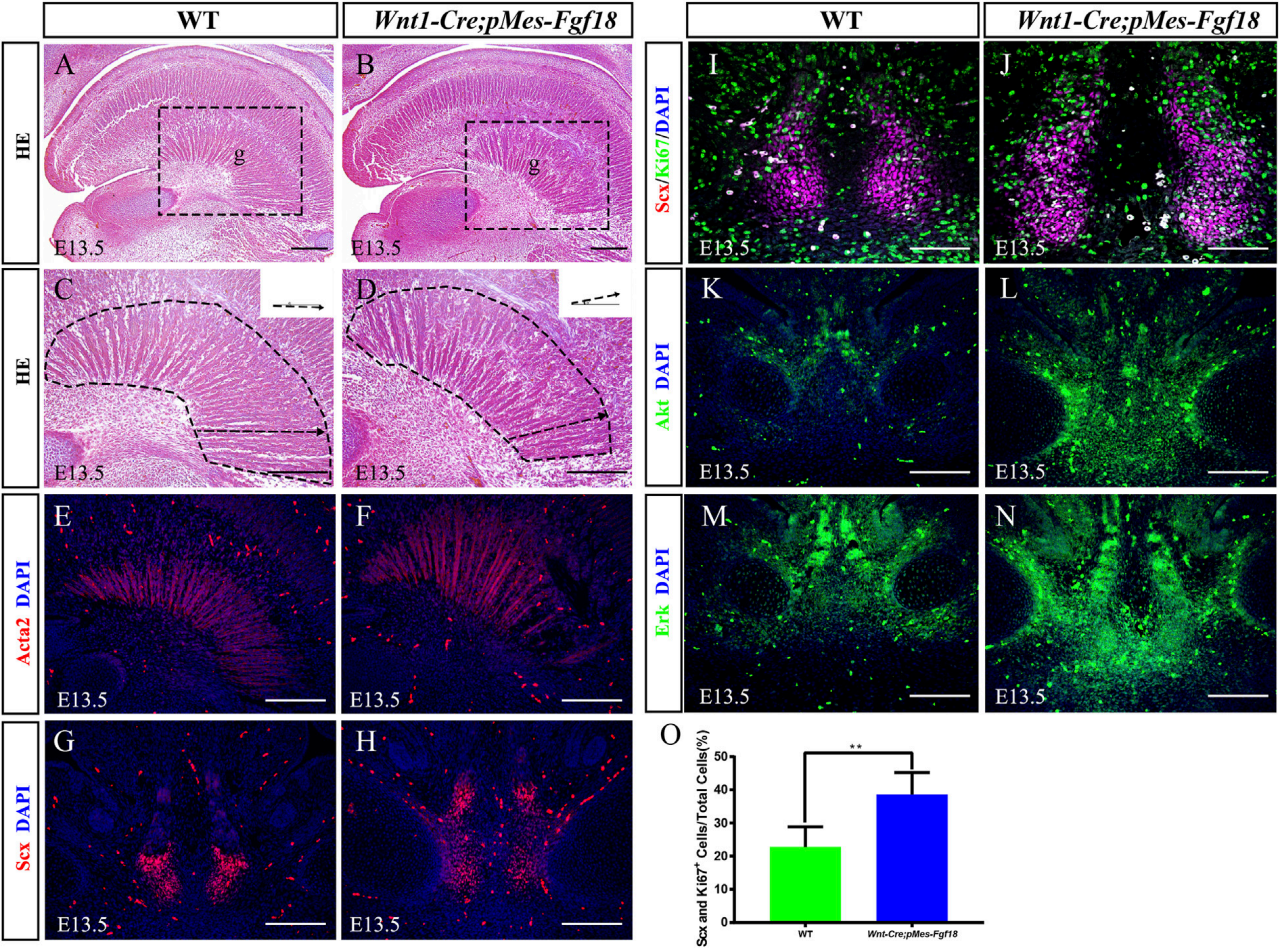
Overexpression of *Fgf18* changed the direction of genioglossus muscle contraction. **(A–D)** Azon staining of sagittal sections of the genioglossus muscle in mice at E13.5. **(E, F)** Immunostaining with antibodies against Acta2 was performed on the sagittal sections of the E13.5 mouse embryos tongue. **(G, H)** Immunostaining with antibodies against Scx was performed on the tongue of mouse embryos at E13.5. **(I, J)** Immunostaining was performed on genioglossus muscle attachment sites of E13.5 mouse embryos using antibodies against Scx and Ki67. **(K–N)** Immunostaining with antibodies against Akt and Erk was performed on the tongue of mouse embryos at E13.5. **(O)** Quantification analysis of the percentage of Scx and Ki67 double-positive cells in the tongue tendon cells. m: Meckel cartilage, g: genioglossus, Scale bar = 200 µm **(A–H, L–O)**, Scale bars = 100 μm **(I, J)**. **p < 0.01.

The genioglossus muscle, located in the face, is crucial for controlling tongue movement (Kouskoura et al., 2016). To explore the impact of genioglossus contraction direction on tongue movement, we performed histological analysis on the tongues of E13.5 embryos. Our findings revealed that, in contrast to controls, the fibers of the genioglossus in *Wnt1-Cre;pMes-Fgf18* mice displayed a distinct shift in orientation, adopting a more acute angle relative to the horizontal plane (Figures 4A–D). Immunofluorescence staining of Acta2, a marker of myofibroblasts, further supports the observed alteration in genioglossus fiber orientation (Figures 4E, F) (Supplementary Figure S3G). To determine whether the altered direction of genioglossus muscle contraction was attributed to abnormal genioglossus muscle attachment, we evaluated the expression of Scx, a marker for tendons, in E13.5 *Wnt1-Cre;pMes-Fgf18* mice. The findings revealed a significant increase in the number of Scx-positive cells in the tongues of *Wnt1-Cre;pMes-Fgf18* mice compared to control animals (Figures 4G, H).

To further investigate the underlying mechanism responsible for the elevated Scx^+^ cell population, we conducted an analysis of cell proliferation and survival in the tongue at E13.5. Immunofluorescence staining revealed a significant increase in the number of Ki67 and Scx double-positive cells in *Wnt1-Cre;pMes-Fgf18* mice at E13.5, while apoptosis levels remained unaltered (Figures 4I, J, O) (Supplementary Figures S3E, F, K). Previous research has suggested that FGF signaling plays a crucial role in the development, repair, and injury of tendons (Eloy-Trinquet et al., 2009). To investigate the influence of overdosed FGF18 on the proliferation of tongue tendon cells, we evaluated the expression of FGF downstream signaling molecules Akt and Erk in E13.5 *Wnt1-Cre;pMes-Fgf18* mice. Immunofluorescence analysis indicated that *Wnt1-Cre;pMes-Fgf18* mice exhibited elevated protein levels of Akt and Erk in tongue tendon cells compared to the control group (Figures 4K–N) (Supplementary Figure S3H). In summary, overexpression of *Fgf18* increases the proliferation of tongue tendon cells and modulates the direction of genoglossus muscle contraction by upregulating the expression of signaling molecules such as Akt and Erk.

### 3.3 Excessive *Fgf18* signaling in CNCCs led to mandibular deformation

**FIGURE 5:**
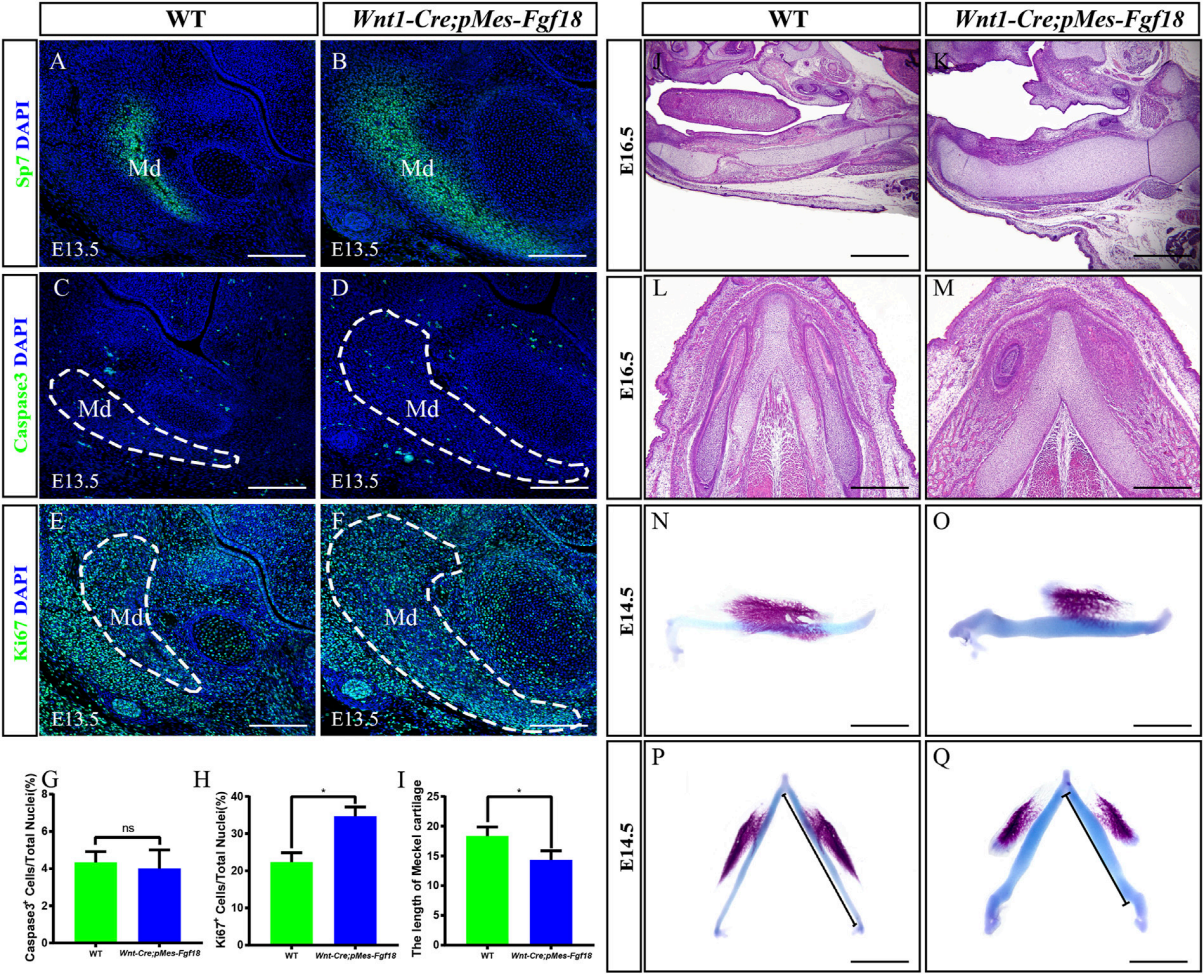
*Wnt1-Cre;pMes-Fgf18* embryos exhibited severely hypoplastic Meckel’s cartilages. **(A–F)** Immunostaining was performed on coronal sections of the mandible at E13.5 with antibodies against Sp7 **(A,B)**, Caspase 3 **(C,D)**, and Ki67 **(E,F)**. **(G,H)** Quantification analysis of the percentage of Caspase 3-positive cells and Ki67-positive cells in the mandible at E13.5. **(I)** Statistical analysis of the length of Meckel’s cartilage. **(J–M)** Hematoxylin and eosin (HE) staining of Meckel’s cartilage of embryos at E16.5. **(N–Q)** The top and side macroscopic views of the lower jaw of mouse embryos at E14.5, following Alcian Blue-Alizarin Red staining. Scale bar = 200 µm **(A–F)**, Scale bar = 400 µm **(J–Q)**, *p < 0.05.

Mandibular dysplasia is a primary etiology of cleft palate. We conducted a comprehensive investigation on the development of the mandible in *Wnt1-Cre;pMes-Fgf18* mice during the stages of E14.5 to P0. Osteogenic staining revealed that the total mandibular length of *Wnt1-Cre;pMes-Fgf18* mice were significantly reduced, and other regions such as the condylar process, condylar cartilage, and angular process also exhibited abnormalities (Supplementary Figures S4A–H). Histological analysis revealed a significant increase in the sectional area of the mandibular coronal and Meckel’s cartilage in *Wnt1-Cre;pMes-Fgf18* mice at E14.5 to E16.5, as compared to the control group (Supplementary Figures S4I–N). Consistent with increased coronal area of the mandible phenotype, immunostaining for Sp7, a marker for osteogenic progenitor cells, revealed a significant increase in the number of positive cells in *Wnt1-Cre;pMes-Fgf18* mice (Figures 5A, B). Immunostaining for Ki67 and Caspase3 revealed that the number of apoptotic cells in mutants was similar to that of controls, however, the percentage of Ki67-positive cells was significantly higher in the mandible of *Wnt1-Cre;pMes-Fgf18* mice at E13.5 (Figures 5C–H), suggesting that the increased mandibular size in the coronal plane is at least partially due to an increase in proliferation in *Wnt1-Cre;pMes-Fgf18* embryos.

Previous research has indicated that abnormalities in Meckel’s cartilage can result in defects in the development and growth of the mandible (Biosse Duplan et al., 2016). To explore Meckel’s cartilage phenotype in *Wnt1-Cre;pMes-Fgf18* mice, we conducted a histological examination on embryonic sections ranging from E14.5 to E16.5. Our findings showed that the degeneration in the cartilage’s middle region is disrupted in mutants (Figures 5J–M). Additionally, Meckel’s cartilage in these mice exhibits a decreased length and is severely distorted (Figures 5I, N–Q). Immunofluorescence analysis of Sox9 expression, a critical gene for chondrocyte fate determination, revealed a significant increase in Sox9-positive cells in the Meckel’s cartilages of E13.5 *Wnt1-Cre;pMes-Fgf18* embryos when compared to controls (Supplementary Figures S5A, B). Furthermore, Ki67 staining indicated an increase in cell proliferation in mutant embryos’ Meckel’s cartilages, while the rate of cell apoptosis in *Wnt1-Cre;pMes-Fgf18* mice was similar to that of controls (Supplementary Figures S5C–H). These findings suggest that the deformation of Meckel’s cartilages is likely linked to the substantial rise in cell proliferation within Sox9-positive cells.

### 3.4 Conditional activation of *Fgf18* induced condylar cartilage defects

**FIGURE 6:**
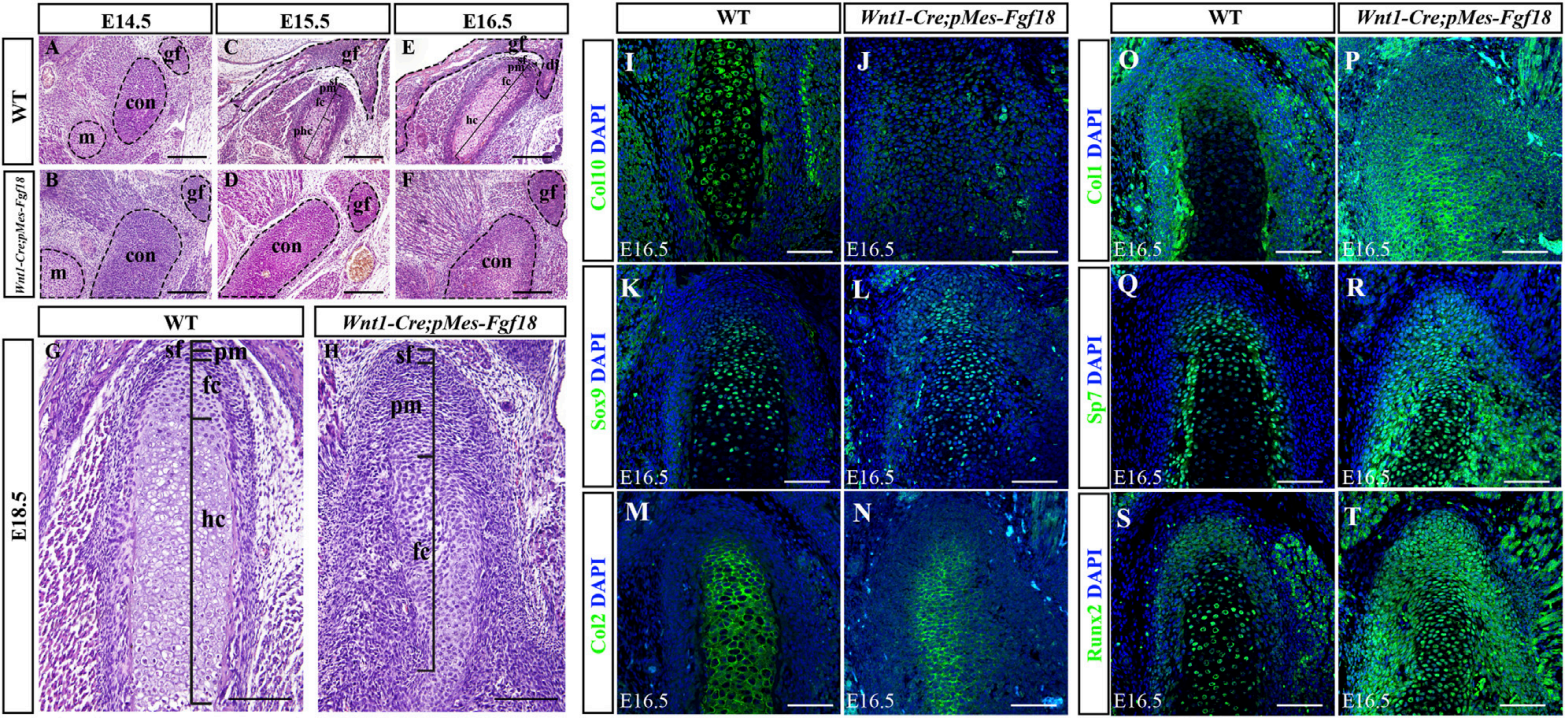
Conditional activation of *Fgf18* resulted in condylar cartilage defects. **(A–F)** Hematoxylin and eosin (HE) stained the condyles of mouse embryos at E14.5∼E18.5. **(I–T)** Immunostaining assays were performed to detect the expression of Col10, Sox9, Col2, Col1, Sp7, and Runx2 proteins in the condylar of mice at E16.5. sf: superficial layer, pm: polymorphic, fc: flattened chondrocyte zone, hc: hypertrophic chondrocyte zone, gf: glenoid fossa, con: condylar cartilage, m: Meckel cartilage, Scale bar = 200 µm **(A–H)**, Scale bar = 100 µm **(I–T)**.

The condylar cartilage, undergoing endochondral ossification, is crucial for mandible growth and development. Defects in this cartilage can cause dental and facial issues like mandibular hypoplasia and dysmorphogenesis (Xu et al., 2022). Histological analyses revealed that during the initial stages of condylar primordia formation at E14.5, the size of the condyle in *Wnt1-Cre;pMes-Fgf18* mice was noticeably larger than that of the control group (Figures 6A, B). This suggested that alterations in the condylar cartilage may play a crucial role in the development of mandibular dysplasia. During the embryonic stages of E15.5 to E18.5, the chondrocytes in the control mice exhibited a well-organized structure, comprising of a superficial cell layer, a polymorphic progenitor cell layer, a zone of flattened chondrocytes, and a zone of hypertrophic chondrocytes that extended from the superficial to the deep cartilage of the condyle. In contrast, *Wnt1-Cre;pMes-Fgf18* mice demonstrated cellular derangement in the condyle (Figures 6C–H). Furthermore, Azan staining results indicated that the intensity of anilin blue staining is significantly lower in the condylar cartilage of *Wnt1-Cre;pMes-Fgf18* mice (Supplementary Figure S6), suggesting that collagen expression was reduced in mutant mice, which is probably caused by changes in cell types.

To evaluate the alterations in cell types and their distribution within the condylar cartilage of *Wnt1-Cre;pMes-Fgf18* mice, we utilized immunofluorescence analysis to identify distinct cell markers. Our findings demonstrate that collagen type 10 (Col10), a biomarker for hypertrophic chondrocytes, is highly expressed in the hypertrophic zone of control E16.5 mice. Conversely, this expression is significantly reduced in *Wnt1-Cre;pMes-Fgf18* mice, suggesting that hypertrophy is disrupted at this stage (Figures 6I, J). The obtained result implied that the activation of FGF18 signaling may cause chondrocyte hypertrophy defects. Subsequently, we conducted experiments to examine the expression of Sox9, Col2, Col1, and Sp7 in the condylar cartilage of *Wnt1-Cre;pMes-Fgf18* mice during the earlier stages of chondrocyte differentiation, prior to the onset of hypertrophy. The immunofluorescence results indicated that in comparison to the control group, the expressions of Sox9 and Col2 were significantly downregulated in *Wnt1-Cre;pMes-Fgf18* mice, whereas the expressions of Sp7 and Col1 were elevated (Figures 6K–R). These results indicated that overdosed FGF18 signaling may accelerate chondrocyte maturation and differentiation towards hypertrophic condyles, ultimately resulting in disparities in the expression of these markers between the two groups.

Runx2 is a crucial transcription factor for skeletal development and plays a role in determining whether chondrocytes become transient cartilage or permanent cartilage. Immunofluorescence results demonstrated that Runx2 was highly expressed in prehypertrophic chondrocytes, while its expression was significantly reduced in other regions of the control mice’s condylar cartilage. Interestingly, in the *Wnt1-Cre;pMes-Fgf18* mice, we observed a significant increase in the number of Runx2-positive cells, particularly in the polymorphic layer cells of the condyle (Figures 6S, T). This finding suggests that the differentiation of condylar chondrocytes is disrupted by the abnormal activation of Runx2 in the polymorphic layer cells of *Wnt1-Cre;pMes-Fgf18* mice.

### 3.5 Excessive FGF18 signaling altered cell proliferation and ossification in the condyle

**FIGURE 7:**
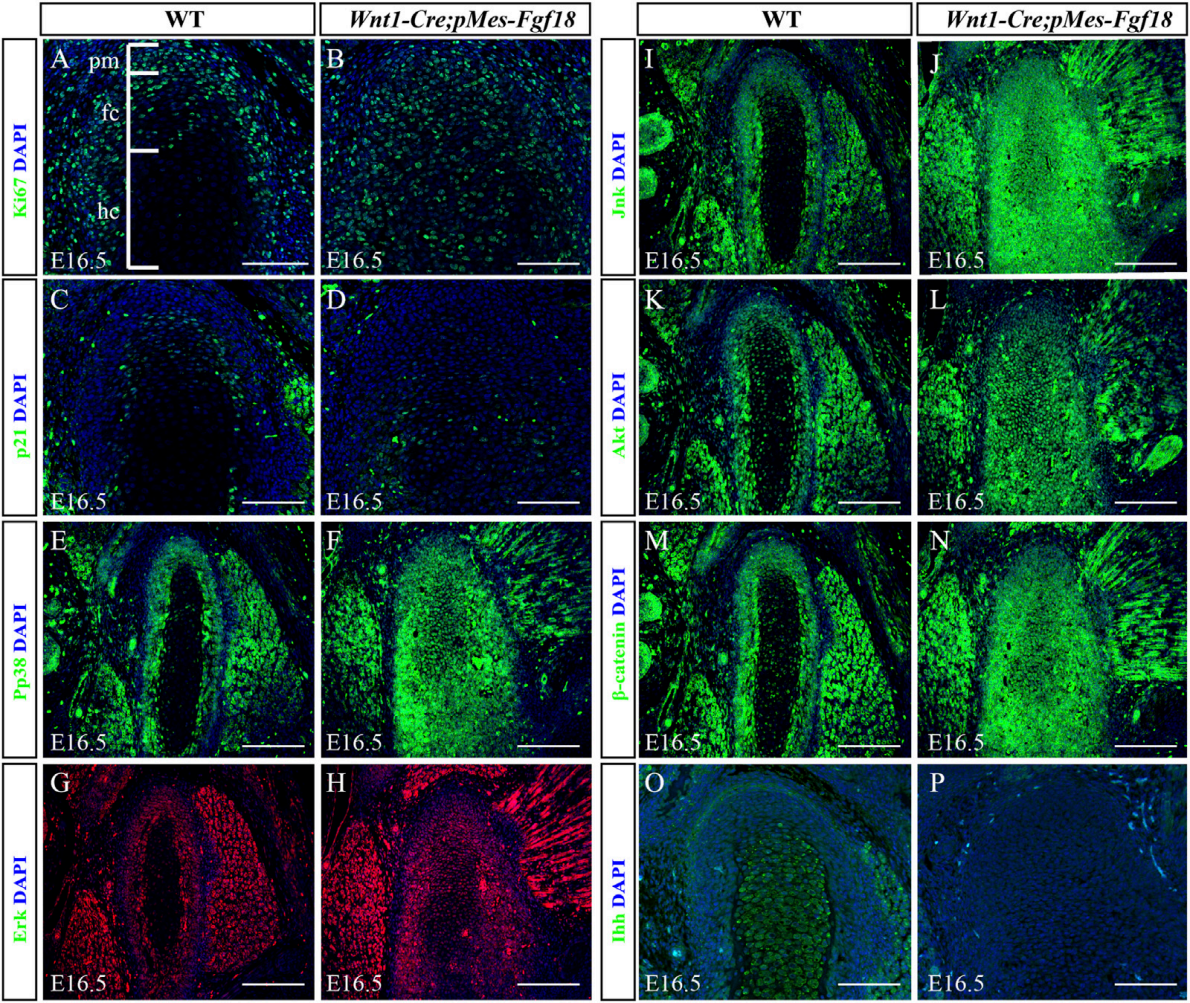
Overexpression of *Fgf18* disrupted downstream signals in condyle. **(A–P)** Immunostaining was performed on the condylar cartilage at E16.5 with antibodies against Ki67 **(A,B)**, p21**(C,D)**, pp38 **(E,F)**, Erk **(G,H)**, Jnk **(I,J)**, Akt **(K, L)**, β-catenin **(M, N)** and Ihh **(O, P)**. pm: polymorphic, fc: flattened chondrocyte zone, hc: hypertrophic chondrocyte zone, Scale bar = 200 µm.

To determine whether *Fgf18* overexpression in CNCCs modulates condylar endochondral ossification by influencing cell proliferation and apoptosis, we evaluated the expression of Ki67 and Caspase3 in the condyle at E16.5. In control mice, Ki67 staining revealed a high presence of proliferating cells in the polymorphic progenitor cell layer and the zone of flattened chondrocytes. Conversely, in *Wnt1-Cre;pMes-Fgf18* mice, there was a significant increase in Ki67-positive cells throughout the entire condylar cartilage (Figures 7A, B). However, the Caspase3 assay showed no significant difference in cell apoptosis in the condyle of *Wnt1-Cre;pMes-Fgf18* mice compared to control mice (Supplementary Figure S7). Previous research has demonstrated that FGF signaling promotes cell proliferation by suppressing p21 expression through activation of the Akt signaling pathway (Lu et al., 2020). To explore whether FGF18 signaling promoted chondrocyte proliferation in the condyle by inhibiting p21 expression, we evaluated the expression levels of p21 in the condyle of *Wnt1-Cre;pMes-Fgf18* mice. Our findings revealed that the expression of p21 was significantly reduced in the condyle of these mice compared to control mice at E16.5 (Figures 7C, D). These findings suggest that the overexpression of *Fgf18* disrupts chondrocyte proliferation, partly due to the abnormal distribution of p21 in the condylar cartilage.

To elucidate the fundamental process of abnormal endochondral ossification, we examined the expression of crucial proteins involved in the FGF signaling pathway within the condylar process of *Wnt1-Cre;pMes-Fgf18* mice. The results indicated that phosphor-p38 and phosphor-Erk1/2 were primarily expressed in the surface cell layer and polymorphic progenitor cell layer of the E16.5 wild-type mouse condyle. However, in mutant mice, their expression was observed to extend to the zone of flattened chondrocytes. Furthermore, most of these proteins were found to be localized in the nucleus, indicating their activation (Figures 7E–H). However, despite the presence of phosphor-JNK in the condyle of the mutant mice, its expression range was expanded to the zone of flattened chondrocytes. Nevertheless, it primarily localized in the cytoplasm, indicating an inactive state (Figures 7I–L). When compared to wild-type mice, the condyle of *Wnt1-Cre;pMes-Fgf18* mice displayed phosphorylation of Akt in almost all cells, indicating that FGF18 may stimulate the proliferation of pre-flattened chondrocytes by activating the Akt signaling pathway (Figures 7K, L).

The Wnt and Hedgehog (Hh) signaling pathways play crucial roles in the development and growth of organs and tissues, as they regulate cell proliferation, differentiation, and migration (Guzzetta et al., 2020; Oichi et al., 2020). To investigate the effect of overdosed Fgf18 in CNCCs on Wnt and Hh signaling, we examined the expression of β-catenin and Ihh in condyle at E16.5. In comparison to wild-type mice, the condyle of *Wnt1-Cre;pMes-Fgf18* mice exhibited a significant increase in β-catenin, particularly in the area where flattened chondrocytes. This suggests that the canonical Wnt signaling pathway is activated in the condyle of these mutant mice (Figures 7M, N). However, when compared to the control group, the expression of Ihh was noticeably reduced in the condylar cartilage of *Wnt1-Cre;pMes-Fgf18* mice. Ihh expression was robust in the hypertrophic chondrocyte region of the condyle, with no detectable expression observed in the polymorphic progenitor layer of wild-type mice. (Figures 7O, P). These findings suggest that Hh signaling may facilitate the differentiation of flat chondrocytes into pre-hypertrophic chondrocytes. Together, excessive FGF18 in CNCCs induced the abnormality of FGF-related signals and disturbed the normal development of condyle.

## 4 Discussion

PRS is a congenital craniofacial structural defect characterized by mandibular hypoplasia, glossoptosis, cleft palate, and upper airway obstruction (Cote et al., 2015). While the underlying pathogenesis of PRS remains to be fully understood, it is believed that the core problems include failure of mandibular outgrowth or muscle defect with incomplete tongue descent (Tan et al., 2013; Juge et al., 2022). Previous research has shown that conditional knockout of *Fgf18* in CNCCs results in mice with cleft palate and micrognathism (Yue et al., 2021). To further explore the mechanisms of FGF18, we utilized a *Wnt1-Cre;pMes-Fgf18* mouse model to specifically activate the conditional *Fgf18* transgenic allele in CNCCs. Our findings reveal that *Wnt1-Cre;pMes-Fgf18* mice exhibited cleft palate, mandibular deformation, and tongue malposition, indicating that a precise equilibrium of endogenous FGF18 signaling is essential for normal craniofacial development. Furthermore, an *in vitro* assay of roller culture showed that mutant palatal shelves elevate normally when the tongue and mandible are removed, suggesting that FGF18 is not a crucial intrinsic factor for regulating palate elevation. This is consistent with previous research that demonstrated that the specific knockout of *Fgf18* in CNCCs does not impact palatal shelf elevation and fusion (Yue et al., 2021). We propose that the FGF18 paradox should not be solely determined by the signaling amplitude, but rather by the cascade that FGF18 transactivates. Here, we have successfully generated an animal model of *Wnt1-Cre;pMes-Fgf18* mice that exhibit a phenotype similar to the human PRS, providing a valuable tool for further elucidating its pathogenesis. However, it is noteworthy that concerns have been raised about the Wnt1-Cre strain, which is widely used, possessing the capability to function as a general gene delete (Dinsmore et al., 2022). Hence, caution and meticulousness are necessary when employing this strain in research.

The mandibular cartilage is known to play a crucial role in the growth and development of the mandible (Xu et al., 2022; Zhang et al., 2022). Our observations revealed that the mandibular morphology of *Wnt1-Cre;pMes-Fgf18* mice was severely shortened, with noticeable abnormalities in Meckel’s cartilage and deformities in condylar cartilage. The defects observed are consistent with previous research reports, which have demonstrated that the overexpression of *Fgf10* or gain-of-function mutations in FGFR3 can lead to changes in the size and shape of Meckel’s cartilage and abnormal condyle, ultimately resulting in mandibular dysmorphogenesis (Mina et al., 2007; Terao et al., 2011). The normal morphogenesis of the mandible relies on two types of ossification: intramembranous ossification and endochondral ossification (Sugito et al., 2011). Previous studies have shown that congenital deficiency of FGF18 or FGFR3 led to similar expansion of growth plates in fetal mice, and the addition of FGF18 can stimulate the repair of damaged cartilage by enhancing proliferation and matrix production (Davidson et al., 2005; Moore et al., 2005). The expression of Sp7 in the mandibular primordium of E13.5 *Wnt1-Cre;pMes-Fgf18* mice was significantly upregulated, indicating that the FGF18 signaling pathway has a crucial impact on the early osteogenic differentiation program. However, the precise impact of FGF18 overexpression on earlier embryonic stages prior to E13.5 remains enigmatic and warrants further investigation.

Studies have shown that FGF plays a crucial role in embryonic development and adult tissue homeostasis by regulating multiple cellular activities such as proliferation, survival, migration, differentiation, and angiogenesis through the modulation of Akt, Erk, P38, p21, Jnk, Wnt, Ihh, and Runx2 signaling pathways (Chen et al., 2017; Zhai et al., 2017; Yao et al., 2019; Hayes et al., 2022). Our findings indicate that overexpression of *Fgf18* in CNCCs disrupts the downstream signals, which subsequently causes abnormalities in cell proliferation and cartilage ossification. These modifications result in alterations in the direction of genioglossus muscle contraction and mandibular deformation. Akt and Erk are crucial components of the BMP, TGFβ, FGF, and EGF signaling pathways. Studies have revealed that mice with disabled ERK or BMP2 signaling in neural crest cells display a phenotype that is comparable to human PRS (Parada et al., 2015; Chen et al., 2019). Additionally, the osteogenic differentiation effects of FGF18 are confirmed in part by increased levels of BMP2 and BMP4 (Jeon et al., 2012; Nagayama et al., 2013). Paradoxically, conditional overexpression of Bmp4 in CNCCs results in a shorter, more pointed mandible compared to controls (Bonilla-Claudio et al., 2012). Meanwhile, we observed abnormalities in the temporomandibular joint (TMJ) of *Wnt1-Cre;pMes-Fgf18 mice.* In comparison to wild-type mice, mutant mice exhibited a suppression in glenoid fossa differentiation, and the articular disc structure was absent at E16.5 (Figure 6). Ihh plays an important role in promoting the development of TMJ (Lu et al., 2022). Our findings indicate that an overdosed FGF18 hinders the expression of Ihh in condylar cartilage, which may be the cause of abnormal TMJ development in mutant mice. The tissue-specific defects observed can be attributed to the unique patterns of FGF18 receptor expression in each tissue, which explains the distinct phenotypic outcomes observed in different tissues when FGF18 is dysregulated. Additionally, the intricate interplay among downstream signaling pathways, such as Akt, Erk, p38, β-catenin, Ihh, Runx2, and p21, is crucial in determining the tissue-specific phenotypic outcomes observed upon dysregulation of FGF18. Given that FGF18 is a secreted protein, its overexpression can potentially disrupt the delicate interaction among different organs. Altered FGF18 expression might affect the endocrine system, thereby modulating hormone secretion and regulation. The specific mechanism underlying the tissue-specific response to FGF18 dysregulation caused by this complex network of signaling molecules remains unclear. Hence, further research is needed to understand the precise mechanisms by which FGF18 regulates these processes and how it contributes to the development of local craniofacial structures.

In summary, our study has shown that overexpression of Fgf18 signaling in CNCCs disrupts the development and growth of the mandible, leading to structural anomalies in the Meckel’s and condylar cartilages. These anomalies are likely caused by significant increases in cell proliferation, defective cell differentiation, and abnormal distribution of correlation signals in the orofacial regions. These findings enhance our understanding of the potential mechanisms underlying the Pierre Robin sequence. However, further research is necessary to fully comprehend the role of FGF18 in craniofacial development and to identify potential therapeutic targets for treating craniofacial disorders caused by FGF18 signaling dysregulation.

## Data Availability

The datasets presented in this study can be found in online repositories. The names of the repository/repositories and accession number(s) can be found in the article/Supplementary Material.
