# Supplementary material for: Overexpression of Fgf18 in cranial neural crest cells recapitulates Pierre Robin sequence in mice
Source: Front Cell Dev Biol. 2024 Apr 17;12:1376814. doi: 10.3389/fcell.2024.1376814 (PMC11061347; doi:10.3389/fcell.2024.1376814)
Supplement: Supplementary file 1 [file Image1.pdf]

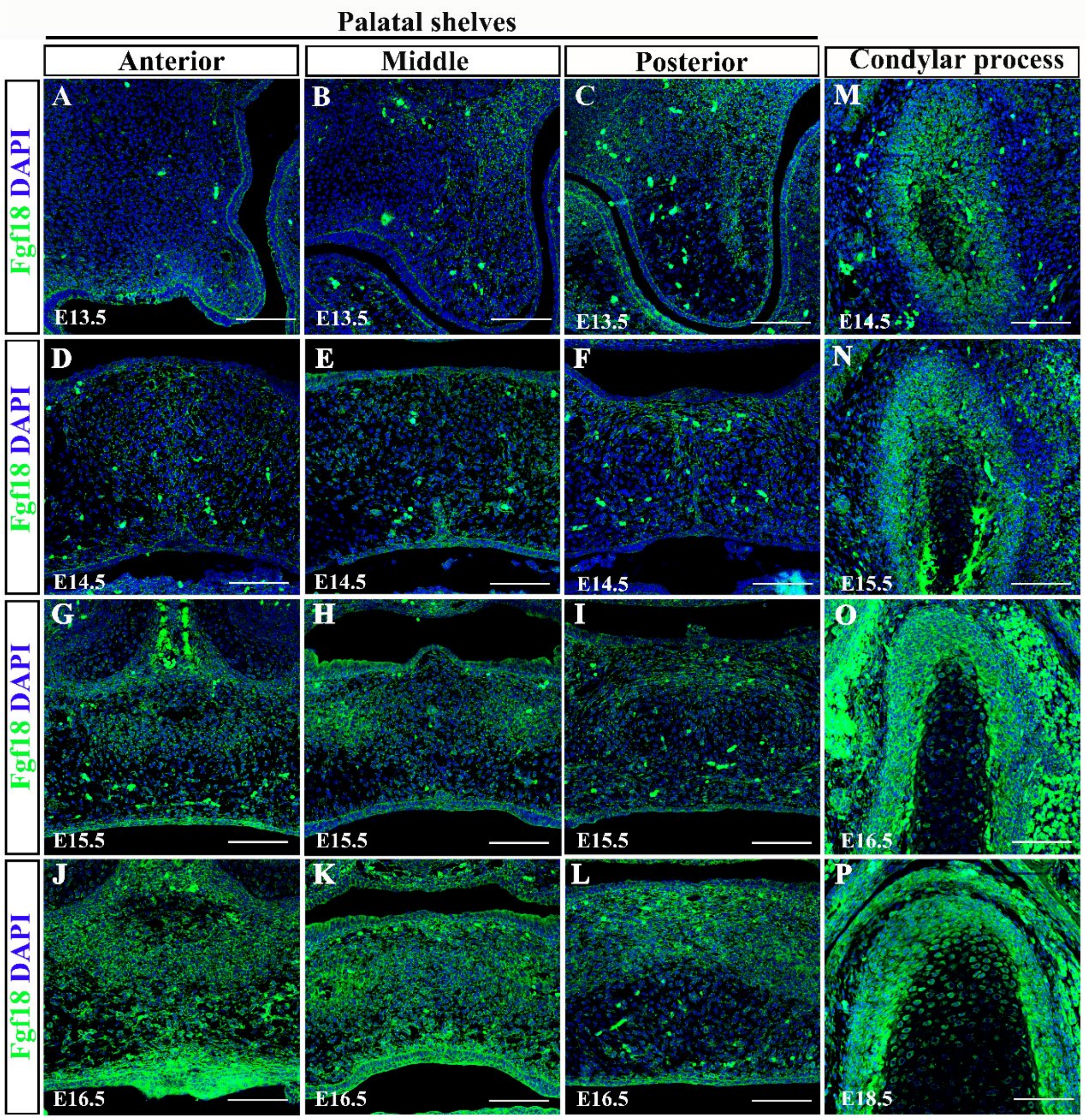

Supplementary Figure 1. Expression pattern of FGF18 in the craniofacial region of mouse embryos. (A-L) Immunostaining of FGF18 on anterior, middle, and posterior palatal frame at E13.5 ~ E16.5. (M-P) Immunostaining of FGF18 in the condyle at E14.5 ~ E18.5. Scale bars = 100 μm.

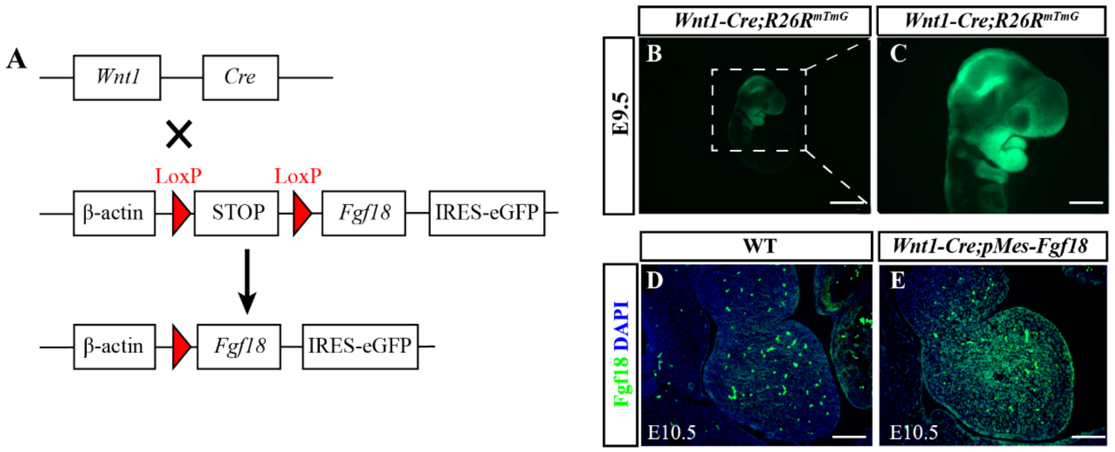

Supplementary Figure 2. Construction and validation of *Fgf18*-specific overexpression in mice. (A) Breeding strategy for conditional *Fgf18* overexpression. (B, C) Fluorometric analysis of *Wnt1-Cre; R26R^{mTmG}* mice at E9.5. (D, E) Immunostaining of FGF18 in the branchial arch of E10.5 mice. Scale bar = 1000 μm (B), Scale bar = 500 μm (C), Scale bar = 200 μm (D, E).

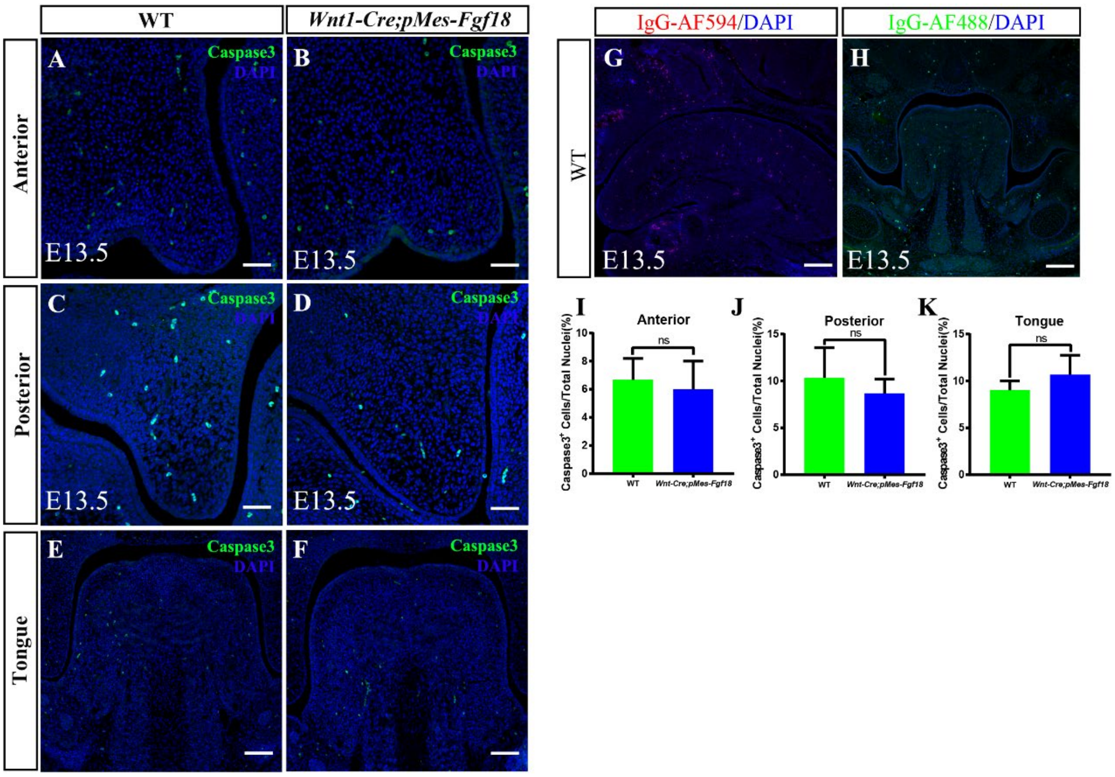

Supplementary Figure 3. Cell apoptosis was unaffected in palatal shelves and tongue of *Wnt1-Cre; pMes-Fgf18* mice. (A-F) Immunostaining with antibodies against Caspase 3 was performed on the palatal shelves and tongue of mouse embryos at E13.5. (G) Immunostaining with antibodies against IgG-AF594 was performed on the sagittal sections of the E13.5 mouse embryos tongue. (H) Immunostaining with antibodies against IgG-AF488 was performed on the E13.5 mouse embryos tongue. (I-K) Quantitative analysis of the cell apoptosis in the palatal shelves and tongue. Scale bar = 200 μm.

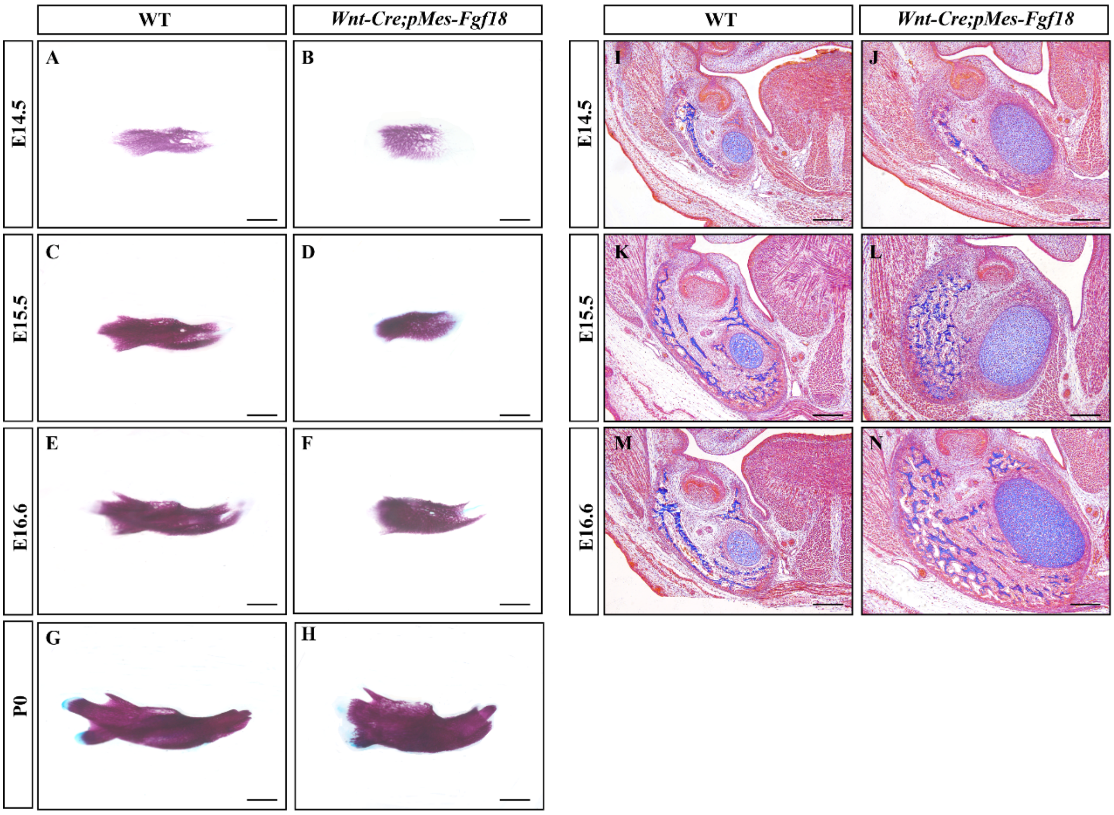

Supplementary Figure 4. *Wnt1-Cre; pMes-Fgf18* mouse embryos showed shorter mandibles. (A-H) Alcian Blue-Alizarin Red staining of the mandibles of the mouse at E14.5 ~ P0. (I-N) Alcian Blue-Alizarin Red staining the coronal sections of the mouse mandible at E14.5 ~ E16.5. Scale bar = 500 μm (A-F), Scale bar = 1000 μm (G, H), Scale bar = 200 μm (I-N).

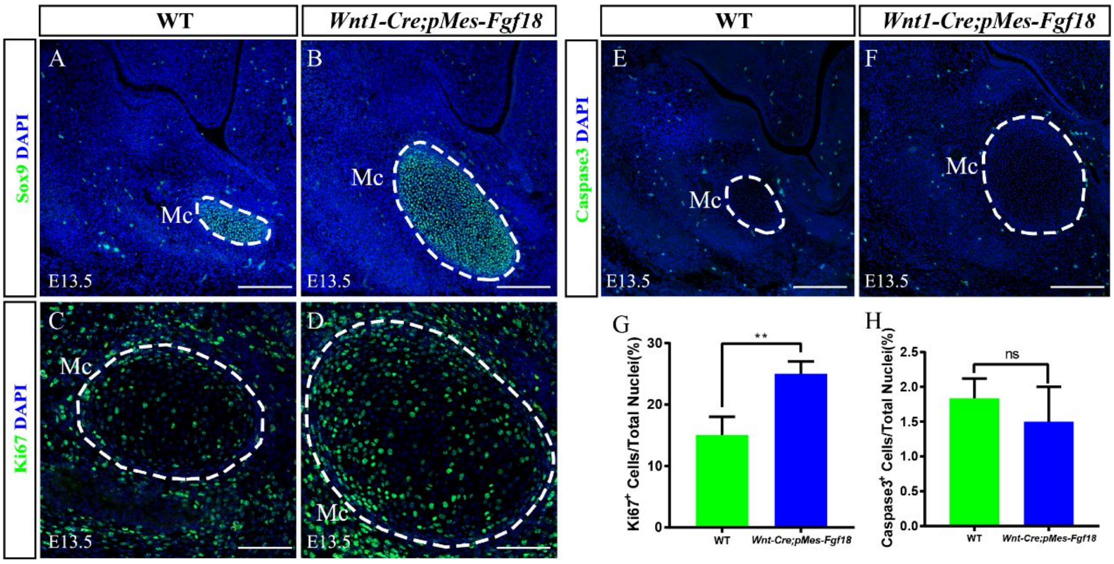

Supplementary Figure 5. Proliferation of chondrocytes was increased in Meckel's cartilages of *Wnt1-Cre;pMes-Fgf18* mice. Immunostaining was performed on the coronal sections of Meckel's cartilages at E13.5 with antibodies against Sox9 (A, B), Caspase 3 (C, D), and Ki67 (E-H). (G, H) Quantification analysis of the percentage of Ki67-positive and Caspase 3-positive cells in the Meckel's cartilage at E13.5. Mc: Meckel's cartilage, Scale bar = 200 μm, **P<0.01.

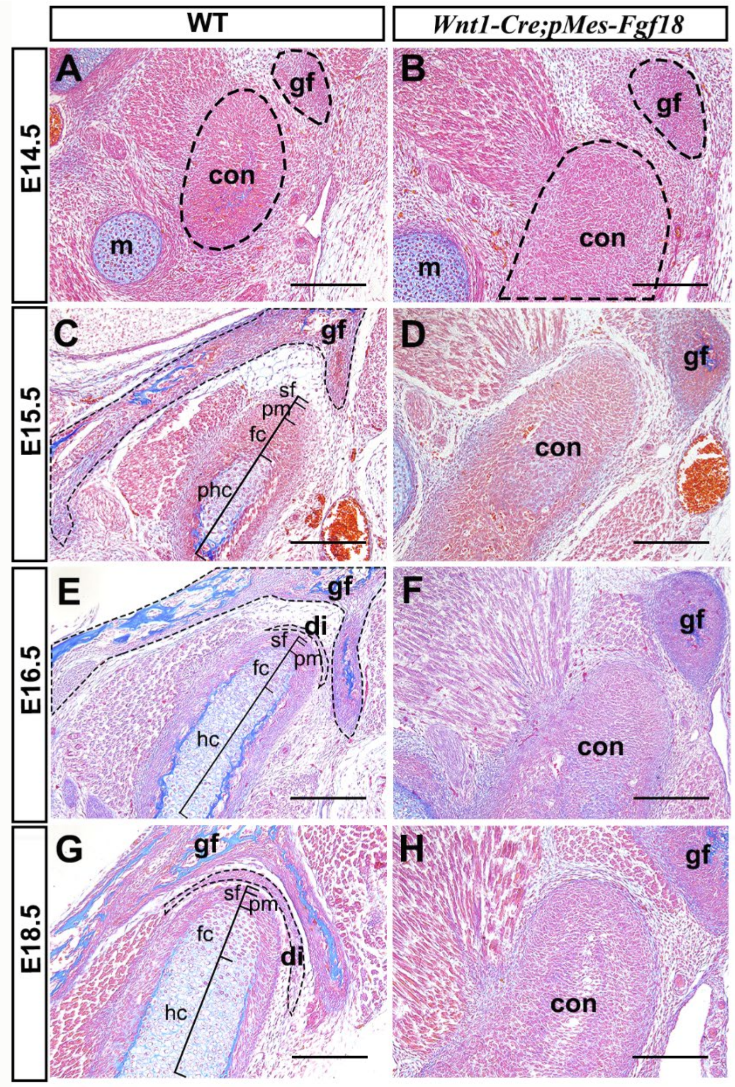

Supplementary Figure 6. Overexpression of *Fgf18* increased proliferation of osteoblast in mandibles. (A-F) Immunostaining was performed on coronal sections of the mandible at E13.5 with antibodies against Sp7 (A, B), Caspase 3 (C, D), and Ki67 (E, F). (G) Quantification analysis of the percentage of Ki67-positive cells in the mandible at E13.5. Scale bar = 200 µm, *P<0.05.

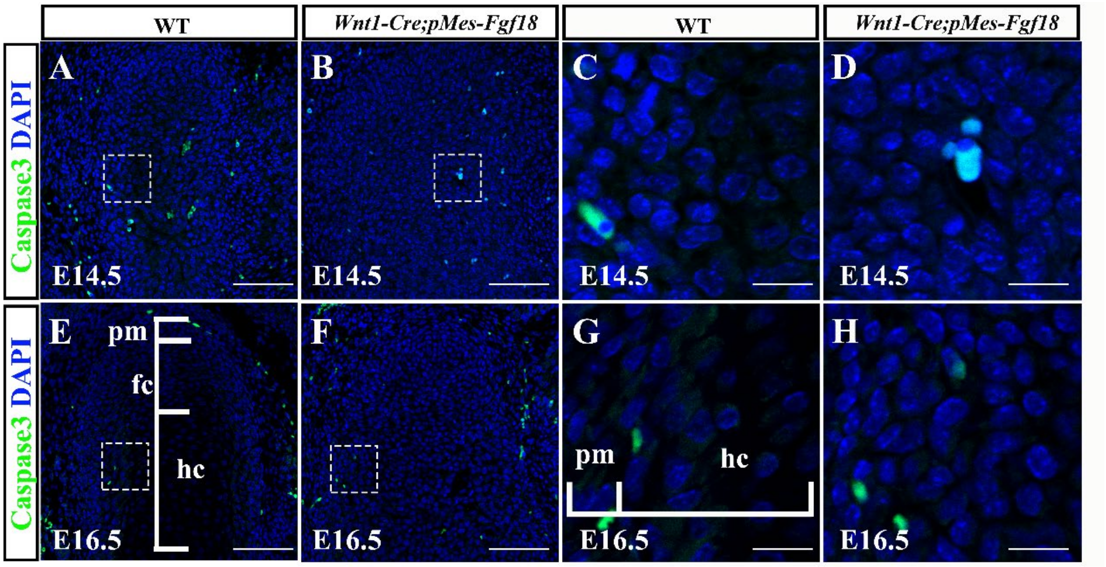

Supplementary Figure 7. Cell apoptosis was unaffected in the condyle of *Wnt1-Cre; pMes-Fgf18* mice. (A-L) The condylar cartilage of E14.5 and E16.5 mouse embryos was immunostained with Caspase 3. pm: polymorphic progenitor cell layer, fc: zone of flattened chondrocytes, hc: zone of hypertrophic chondrocytes, Scale bar = 100 µm(A, B, E, F), Scale bar = 25 µm (C, D, G, H).
